# Comparing the Protective Value of Human and Pet Social Support on Well-Being of Older Adults During COVID-19

**DOI:** 10.1093/geroni/igaa057.3430

**Published:** 2020-12-16

**Authors:** Juliet Sobering, Lisa Brown

**Affiliations:** 1 Palo Alto University, San Jose, California, United States; 2 Palo Alto University, Palo Alto, California, United States

## Abstract

Older adults are vulnerable to particular risk factors that contribute to lower well-being and poorer functioning. With the COVID-19 pandemic, the importance of social support has been highlighted in media reports because of its well-known beneficial effects on overall well-being. However, as adults age, social networks, contacts, and activities naturally decrease. These age-related losses are often difficult, if not impossible, to replace. Pets have recently been recognized as a valuable source of social support for many older adults, providing both physical and psychological benefits through mutual connection and behavioral activation. Previous studies have examined how human social support or pet social support enhance older adults’ well-being (i.e., positive emotions, engagement, relationships, accomplishment, and meaning). However, there is a gap in our scientific knowledge as previous research has not evaluated if pet social support can serve as a protective factor in the absence of adequate human social support. Current analyses, with 141 older adult participants, suggests that pet owners with a positive attachment to their pet experience higher well-being as pets serve as a coping resource that protects against common life stressors. Similar to human social support, pet social support appears to be a protective factor that also promotes and fosters a sense of well-being in older adults. Support in late life is especially important for families and agencies to be attuned to, especially during a global pandemic.

